# Relationship Between Complex Signal Identification and Non‐Pulmonary Vein Foci

**DOI:** 10.1002/joa3.70230

**Published:** 2025-11-26

**Authors:** Hiroyuki Kono, Kenichi Hiroshima, Keigo Misonou, Koumei Onuki, Maiko Kuroda, Jun Hirokami, Tomonori Katsuki, Rei Kuji, Kengo Korai, Masato Fukunaga, Michio Nagashima, Kenji Ando

**Affiliations:** ^1^ Department of Cardiology Kokura Memorial Hospital Kitakyushu Japan

**Keywords:** atrial fibrillation, CARTO 3, catheter ablation, complex signal identification, non‐PV foci

## Abstract

**Background:**

Pulmonary vein isolation (PVI) is the cornerstone of atrial fibrillation (AF) ablation; however, recurrences often originate from non‐pulmonary vein (non‐PV) foci. The Complex Signal Identification (CSI) algorithm in CARTO 3 assigns electrogram‐fractionation scores (0–10). This study evaluated the feasibility of CSI‐assisted mapping for identifying non‐PV triggers.

**Methods:**

We retrospectively analyzed 23 consecutive patients undergoing first‐time AF ablation between January and June 2024. After PVI, non‐PV triggers were induced using isoproterenol and adenosine triphosphate (ATP). When ectopy was absent, rapid pacing during isoproterenol infusion followed by defibrillation was performed, and ATP testing was repeated. High‐density CSI mapping was conducted during sinus rhythm or high right atrial pacing using system default settings, targeting atrial components after ventricular blanking.

**Results:**

A total of 33 non‐PV foci were localized (20 left atrium [LA], 13 right atrium [RA]). The mean CSI scores of LA and RA foci were 9.0 ± 2.3 and 8.8 ± 2.7, respectively. ROC analysis showed an AUC of 0.917 for discriminating non‐PV foci, with an optimal cutoff of 8.5 (sensitivity 87.9%, specificity 88.3%). At 12 months, arrhythmia‐free survival was 82.4% under symptom‐driven follow‐up. Ablation was selectively performed at the earliest activation and adjacent high‐CSI points, avoiding indiscriminate lesion delivery.

**Conclusions:**

CSI‐assisted mapping provided practical, adjunctive guidance to provocation and activation mapping for non‐PV focus localization. While apparent discrimination was promising, the 8.5 threshold remains exploratory. Larger multicenter studies with standardized CSI settings and systematic post‐ablation assessment are warranted to validate these preliminary findings.

## Graphical abstract

Using the CARTO CSI algorithm, electrogram fractionation (score ≥ 8.5) accurately localized non‐pulmonary vein triggers. CSI‐assisted ablation guided by these high‐complexity sites resulted in 82.4% arrhythmia‐free survival, demonstrating the feasibility of CSI as a selective mapping adjunct comparable to FAAM.

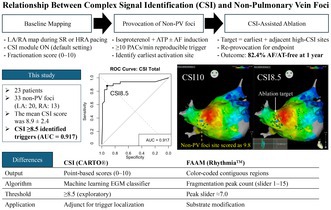

## 1 Introduction

Catheter ablation has become a cornerstone therapy for atrial fibrillation (AF), with pulmonary vein isolation (PVI) established as the principal procedural target [1, 2]. Nevertheless, arrhythmia recurrence following ablation remains frequent. A substantial proportion of these relapses is attributable to ectopic drivers arising outside the pulmonary veins (non‐PV foci), which are increasingly recognized as critical determinants of long‐term outcomes [3, 4]. Previous investigations have suggested that durable suppression of these non‐PV triggers is associated with improved rhythm control, whereas incomplete elimination often predisposes patients to recurrent AF [5, 6, 7, 8].

Technological advances in electroanatomical mapping have facilitated a more detailed characterization of atrial substrates beyond the PVI. The Rhythmia platform (Boston Scientific, Marlborough, MA) incorporates a dedicated algorithm—fractionated atrial activity mapping (FAAM)—that automatically detects electrogram fragmentation (Lumipoint module) [9]. Clinical studies have reported favorable outcomes with FAAM‐guided ablation, with arrhythmia‐free survival rates approaching 90% at 1 year, which surpass those achieved with conventional methods [10].

Building on this concept, the CARTO 3 system (Biosense Webster, Diamond Bar, CA) has recently integrated the Complex Signal Identification (CSI) module. Initially designed for atrial flutter mapping, CSI applies a machine learning‐based model to assign fractionation scores (0–10) to atrial electrograms recorded during sinus rhythm or atrial pacing. Although originally developed for macro‐reentrant circuits, its possible role in AF ablation has drawn increasing attention. Early observations from our group indicated that areas with elevated CSI values often coincided with the sites of earliest activation during the induction of non‐PV triggers, and focal ablation at these locations resulted in arrhythmia termination [11]. However, systematic evidence regarding the utility of CSI‐guided mapping in this setting has been limited.

Accordingly, the present study aimed to assess the performance of CSI‐based mapping for the detection and ablation of non‐PV foci in patients undergoing AF ablation. We hypothesized that integrating automated electrogram fractionation scores with high‐density mapping would provide a reproducible and practical strategy to guide lesion placement beyond the pulmonary veins. To our knowledge, this represents the first systematic evaluation of CSI‐guided ablation for non‐PV triggers in AF.

## 2 Methods

### 2.1 Study Population

This retrospective analysis included consecutive patients with symptomatic AF who underwent their first catheter ablation at Kokura Memorial Hospital between January and June 2024. All procedures were performed using the CARTO 3 electroanatomical mapping system (Version 8, Biosense Webster, Diamond Bar, CA). Both paroxysmal and persistent AF cases were enrolled according to conventional definitions: paroxysmal AF was defined as episodes terminating spontaneously within 7 days, whereas persistent AF lasted beyond 7 days or required cardioversion. Long‐standing AF referred to continuous AF for more than 12 months [12]. All patients had failed at least one antiarrhythmic medication. Exclusion criteria were prior AF ablation or previous cardiac surgery. Baseline demographics, arrhythmia history, and comorbidities were collected from medical records. The study protocol was approved by the local ethics committee (approval no. 25070201) and adhered to the Declaration of Helsinki. Owing to the retrospective design, written informed consent was waived.

### 2.2 Electrophysiological Study and Mapping

Ablations were performed under conscious sedation or general anesthesia. Vascular access was obtained through the femoral veins. A 20‐pole catheter (BeeAT, Japan Lifeline, Tokyo, Japan) was positioned in the coronary sinus for reference, and left atrial (LA) access was achieved with transseptal puncture. A 3.5‐mm irrigated‐tip ablation catheter and a high‐density mapping catheter (Octaray, 3‐3‐3 configuration; Biosense Webster) were introduced into the LA. Electroanatomical maps were created during sinus rhythm or high right atrial pacing using the CARTO 3 system. Pulmonary vein isolation (PVI) was performed in all patients, with bidirectional block verified by a circular mapping catheter. The Complex Signal Identification (CSI) module was then activated to automatically highlight regions of electrogram fractionation on the 3D map. High‐density LA and RA maps were created in sinus rhythm or during high right‐atrial pacing (target cycle length ≈750–800 ms when feasible). CSI (CARTO v8) was applied using system default parameters. Analysis targeted atrial electrograms after ventricular blanking within the window of interest. Bipolar amplitude range, minimum complex duration, and minimum inter‐deflection interval were not manually altered. CSI scores (0–10) were displayed as point tags without surface interpolation.

### 2.3 Provocation and Identification of Non‐PV Triggers

After pulmonary vein isolation, non‐PV triggers were assessed using a standardized provocation protocol. When spontaneous ectopy was observed, electrode catheters were used to map the origin. If ectopy was absent, adenosine triphosphate (20–60 mg) was administered intravenously during continuous isoproterenol infusion (1–10 μg/kg/min) to induce non‐PV triggers [13, 14]. If AF was not provoked by these agents, rapid atrial pacing (50 ms, 30 mA, 5 s) at the high right atrium during isoproterenol infusion was performed, followed by intracardiac defibrillation to restore sinus rhythm and reassessment for recurrent triggers.

Triggers were classified as sustained (> 30 s of AF initiation) or non‐sustained (≥ 10 PACs/min from a single focus). Potential foci included the LA septum, peri‐mitral region, anterior and posterior LA walls, LAA, interatrial septum, posterior RA, SVC, crista terminalis, and coronary sinus; right atrial mapping was performed when RA‐origin triggers were suspected.

### 2.4 Ablation Strategy With CSI Guidance

The overall procedural workflow—comprising mapping, provocation, and CSI‐assisted ablation—is illustrated in Figure S1. Radiofrequency (RF) energy was delivered with power titrated between 25 and 30 W depending on the site and adjacent structures. SVC triggers were treated by wide‐area encirclement at the SVC–RA junction, with diaphragmatic pacing performed to prevent phrenic nerve injury. For foci on atrial walls or septum, focal RF applications were targeted to the earliest activation site and areas exhibiting complex fractionation. CSI‐guided mapping was used to refine ablation by tagging high‐score regions (typically ≥ 8.5), which were cross‐checked with provoked activation sites before lesion delivery. RF applications were continued until abnormal potentials disappeared and ectopy was no longer inducible. If multiple foci were present, each was mapped and ablated sequentially. Ablation strategy was summarized in Figure S1. Procedural success was defined as non‐inducibility of AF, atrial tachycardia (AT), or repetitive non‐PV ectopy after repeat isoproterenol and ATP provocation. At the end of the procedure, PVI was reconfirmed, and additional lesion sets (e.g., SVC isolation, cavotricuspid isthmus [CTI] block) were validated for bidirectional conduction block.

### 2.5 Post‐Procedure Management and Follow‐Up

Patients were monitored with continuous telemetry for 24–48 h following ablation. Oral anticoagulation was maintained for at least 3 months and thereafter according to individual stroke risk. Antiarrhythmic drugs were resumed during the 90‐day blanking period if early recurrence occurred, and discontinued if no further AF was documented. Follow‐up visits were scheduled at 3, 6, and 12 months, including clinical assessment and 12‐lead ECG. Ambulatory monitoring was arranged for patients with symptoms suggestive of arrhythmia. Recurrence was defined as any atrial tachyarrhythmia lasting ≥ 30 s beyond the 3‐month blanking period, in accordance with current consensus criteria.

### 2.6 Statistical Analysis

Continuous data are presented as mean ± standard deviation. Receiver‐operating characteristic (ROC) analysis was performed to test the discriminatory ability of continuous variables. The area under the ROC curve (AUC) was calculated, and the optimal cutoff was derived using the Youden index. Analyses were performed with R software (version 4.3.0; R Foundation for Statistical Computing, Vienna, Austria).

## 3 Results

### 3.1 Baseline Characteristics

A total of 23 patients were included in this study. The mean age was 69.9 ± 8.9 years, and 52% were male (12 males, 11 females). The average height and body weight were 161 ± 11 cm and 63.3 ± 14.5 kg, respectively, with a mean body mass index (BMI) of 24.4 ± 4.7 kg/m^2^. Comorbid conditions included hypertension in 56.5% (13/23), diabetes mellitus in 17.4% (4/23), cardiomyopathy in 21.7% (5/23), and chronic heart failure in 21.7% (5/23). No patients had a history of stroke or transient ischemic attack, vascular disease, or coronary artery disease. The mean CHADS_2_ score was 1.1 ± 0.9, and the mean CHA_2_DS_2_‐VASc score was 2.6 ± 1.4. Antiarrhythmic drugs were being taken by 4 patients (17.4%). Paroxysmal atrial fibrillation was present in 8 patients (34.8%). (Table 1).

**TABLE 1:** Baseline characteristics of the study population.

Age—year	69.9 ± 8.9
Male/female	12/11
Height ‐cm	161 ± 11
Body Weight ‐kg	63.3 ± 14.5
BMI	24.4 ± 4.7
Antiarrhythmic drugs	4 (17%)
Hypertension	13 (57%)
Diabetes	4 (17%)
Stroke/transient ischemic attack	0
Cardiomyopathy	5 (22%)
Chronic heart failure	5 (22%)
Vascular disease	0
Coronary artery disease	0
CHADS2 score	1.1 ± 0.9
CHA2DS2‐VASc score	2.6 ± 1.4
Type of AF	
Paroxysmal AF	8 (35%)
Persistent AF	6 (26%)
Long‐standing persistent AF	9 (39%)
Echocardiographic parameters	
Ejection fraction ‐%	56.0 ± 14.0
LA dimension ‐mm	44.5 ± 6.9
LA volume/body mass index –mL/m^2^	48.5 ± 15.1

*Note:* Values are expressed as mean ± standard deviation (SD) or *n* (%), unless otherwise indicated. AF indicates atrial fibrillation; BMI, body mass index; CHADS_2_/CHA_2_DS_2_‐VASc, congestive heart failure, hypertension, age ≥ 75 years, diabetes mellitus, stroke or transient ischemic attack, vascular disease, age 65–74 years, and sex category; CHF, congestive heart failure; LA, left atrium; LAA, left atrial appendage.

### 3.2 Procedure Characteristics

PVI was performed in all cases, with CTI block line ablation performed in 5 cases and SVC isolation in 3 cases. The mean procedure time was 131 ± 32 min, and the mean fluoroscopy time was 18.2 ± 7.3 min. Mapping time averaged 12.0 ± 2.6 min in the LA and 8.5 ± 3.6 min in the RA. The mean number of RF applications was 51 ± 15. Acute procedural success—defined as a reduction in non‐PV foci to fewer than 10 occurrences per minute within 1 min of induction—was achieved in all cases. (Table 2).

**TABLE 2:** Procedural characteristics, mapping details, and distribution of non‐pulmonary vein (non‐PV) foci.

Procedure time ‐min	131 ± 32
Fluoroscopy time ‐min	18.2 ± 7.3
Fluoroscopy dose ‐mGy	70 ± 86
Number of RF applications	51 ± 15
Acute success for non‐PV foci ablation	23 (100%)
Procedure	
PVI	23 (100%)
CTI	5 (22%)
SVCI	3 (13%)
Mapping parameters	
LA mapping	*N* = 22
During sinus rhythm	9 (41%)
Cycle length ‐ms	844 ± 98
During HRA pacing	13 (59%)
Cycle length ‐ms	696 ± 85
Mapping Time in LA ‐min	12.0 ± 2.6
Number of points	10 644 ± 5320
RA mapping	*N* = 11
During sinus rhythm	11 (100%)
Cycle length ‐ms	890 ± 123
Mapping Time in RA ‐min	8.5 ± 3.6
Number of points	3649 ± 3628
Multiple non‐PV foci ‐cases	9
Non‐PV foci in LA ‐cases	18 (78%)
Non‐PV foci in RA ‐cases	11 (48%)
Total number of non‐PV foci	33
Sustained trigger	6 (18%)
Non‐sustained trigger	27 (82%)
Location of non‐PV foci	
LA	*N* = 20
LA septum	7
LA anterior	5
LA posterior	0
LA inferior	2
Peri‐mitral annulus	3
LAA ostium	3
RA	*N* = 13
SVC	2
Crista terminalis	3
RA posterior	1
RA septum	6
RA inferior	1
CSI score of non‐PV foci (total)	8.9 ± 2.4
Non‐PV foci in LA	9.0 ± 2.3
Non‐PV foci in RA	8.8 ± 2.7

*Note:* Values are expressed as mean ± SD or *n* (%). Acute success was defined as complete elimination of all non‐PV foci following ablation. Mapping parameters include cycle length, mapping duration, and number of acquired points. Non‐PV foci are categorized as sustained (ectopy initiating AF ≥ 30 s) or non‐sustained (≥ 10 PACs/min from a single focus).

Abbreviations: CTI, cavotricuspid isthmus; LA, indicates left atrium; LAA, left atrial appendage; PVI, pulmonary vein isolation; RA, right atrium; RF, radiofrequency; SVCI, superior vena cava isolation.

### 3.3 Mapping and CSI Preference

**FIGURE 1:**
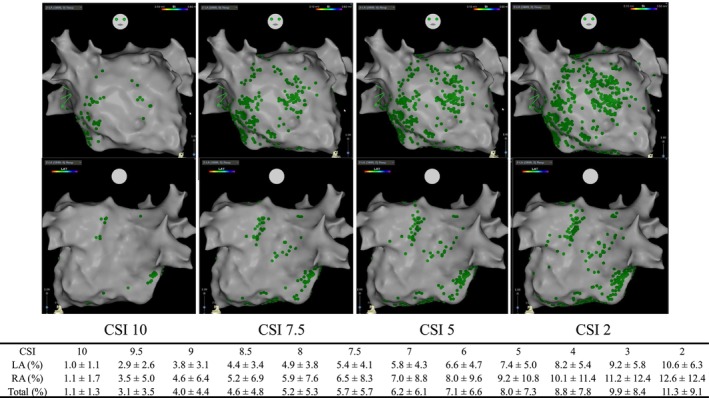
Distribution of fractionated atrial electrograms identified by the Complex Signal Identification (CSI) algorithm at different threshold levels. Representative CARTO 3 electroanatomical maps of the left atrium are shown in anterior–posterior (AP, top) and posterior–anterior (PA, bottom) views for CSI thresholds of 10, 7.5, 5, and 2. Green tags indicate sites of electrogram fractionation that met the corresponding CSI threshold. As the CSI threshold was lowered, a progressive increase in the number of tagged sites was observed. The accompanying table summarizes the proportion of tagged sites in the left and right atria across CSI scores ranging from 10 to 2. Values are presented as mean ± standard deviation. AP, anterior–posterior; CSI, complex signal identification; LA, left atrium; PA, posterior–anterior; RA, right atrium.

Of the 23 enrolled patients, LA CSI analysis suitable for the primary endpoints was available in 22; one patient had an isolated RA non‐PV trigger that was mapped during coronary‐sinus pacing and was therefore excluded from LA CSI analyses. Among the remaining 22 patients, LA mapping was performed during high right‐atrial pacing in 13 and sinus rhythm in 9 (cycle length 757 ± 116 ms). RA mapping was performed in 11 patients with RA‐origin triggers (sinus rhythm; cycle length 890 ± 123 ms). The mean number of mapping points acquired in the LA was 10 644 ± 5320, while in the RA it was 3649 ± 3628. In the LA, 1.0% of mapping points were highlighted at a CSI score of 10, 4.4% at 8.5, 5.4% at 7.5, 7.4% at 5.0, and 10.6% at 2.0. In the RA, 1.1% of points were highlighted at a CSI score of 10, 5.2% at 8.5, 6.5% at 7.5, 9.2% at 5.0, and 12.6% at 2.0. (Figure 1).

### 3.4 Distribution of Non‐PV Foci

A total of 33 non‐pulmonary vein (non‐PV) foci were identified in 23 cases, with 9 cases exhibiting multiple non‐PV foci. Non‐PV foci were detected in the LA in 18 cases and in the RA in 11 cases. Among these, 6 were sustained triggers, while the remaining 27 were non‐sustained. Of the total foci, 20 were located in the LA and 13 in the RA. Within the LA, the most common sites were the septum (7 foci), peri‐mitral region (6 foci), and the anterior wall (5 foci). Two foci were found at the inferior wall of the LA, and no non‐PV triggers were identified on the posterior wall or LAA. The mean CSI score of LA non‐PV triggers was 9.0 ± 2.3. Triggers were classified as sustained when ectopy initiated AF lasting > 30 s and non‐sustained when ≥ 10 PACs/min arose from a single site (see Methods).

**FIGURE 2:**
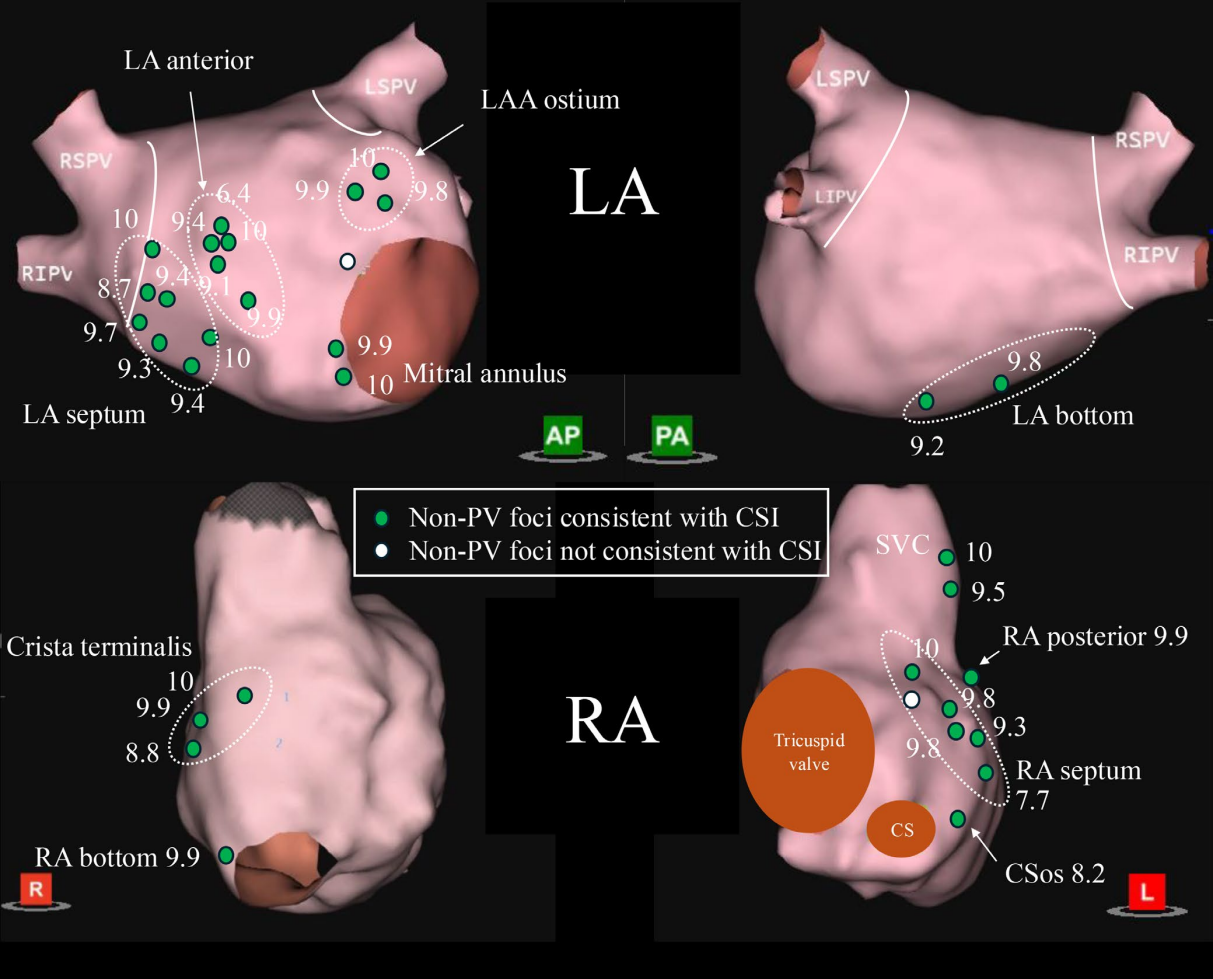
Anatomical distribution of non–pulmonary vein (non‐PV) foci and their consistency with CSI score. Three‐dimensional electroanatomical maps of the left atrium (LA) and right atrium (RA) are shown with the locations of spontaneous non‐PV triggers (green and white circles). Green circles indicate non‐PV foci that were consistent with sites identified by Complex Signal Identification (CSI) mapping, while white circles represent those not overlapping with CSI‐tagged regions. Most non‐PV foci consistent with CSI were located near the LA septum, mitral annulus, crista terminalis, and posterior RA. These findings suggest a spatial correlation between automated electrogram complexity and arrhythmogenic foci. CSI, complex signal identification; LA, left atrium; non‐PV, non–pulmonary vein; RA, right atrium.

In the RA, the septum was the most frequent site (6 foci), followed by the crista terminalis (3 foci), the superior vena cava (SVC; 2 foci), the posterior wall (1 focus), and the inferior wall (1 focus). The mean CSI score of RA non‐PV triggers was 8.8 ± 2.7. (Figure 2) There was one non‐PV focus at the mitral annulus in the left atrium and one at the right atrial septum that did not coincide with CSI‐positive sites.

### 3.5 CSI Score Analysis

ROC curve analysis was performed to evaluate the predictive utility of the CSI score for identifying non‐PV foci. The area under the ROC curve (AUC) was 0.917 for the overall dataset, indicating apparent discriminative capacity. When stratified by atrial chamber, the AUC was slightly higher for LA at 0.924 compared to 0.914 for RA.

**FIGURE 3:**
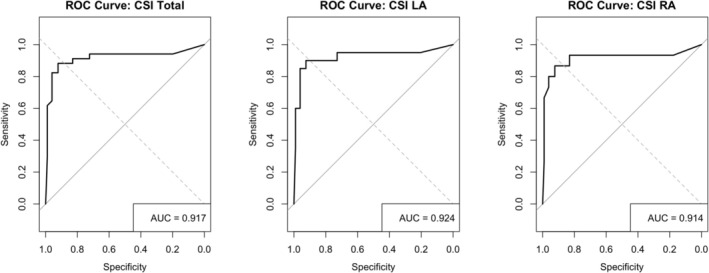
Receiver operating characteristic (ROC) curves for CSI scores in predicting non‐PV triggers. ROC analysis was performed to assess the diagnostic performance of Complex Signal Identification (CSI) scores in identifying non–pulmonary vein (non‐PV) foci. The left panel shows the ROC curve for total CSI score (area under the curve [AUC] = 0.917), while the middle and right panels present separate analyses for the left atrium (LA; AUC = 0.924) and right atrium (RA; AUC = 0.914), respectively. These results demonstrate high accuracy of CSI‐based mapping in both atria for detecting potential non‐PV trigger sites. AUC, area under the curve; CSI, complex signal identification; ROC, receiver operating characteristic; LA, left atrium; non‐PV, non–pulmonary vein; RA, right atrium.

Using a uniform cutoff value of 8.5, the CSI score demonstrated a sensitivity of 87.9% and a specificity of 88.3% for the total cohort, with a corresponding Youden Index of 0.761. When evaluated separately, the left atrium yielded a sensitivity of 90.0% and specificity of 92.4% (Youden Index = 0.824), whereas the right atrium showed a sensitivity of 86.7% and specificity of 92.3% (Youden Index = 0.790). (Figure 3).

### 3.6 Efficacy Outcomes

**FIGURE 4:**
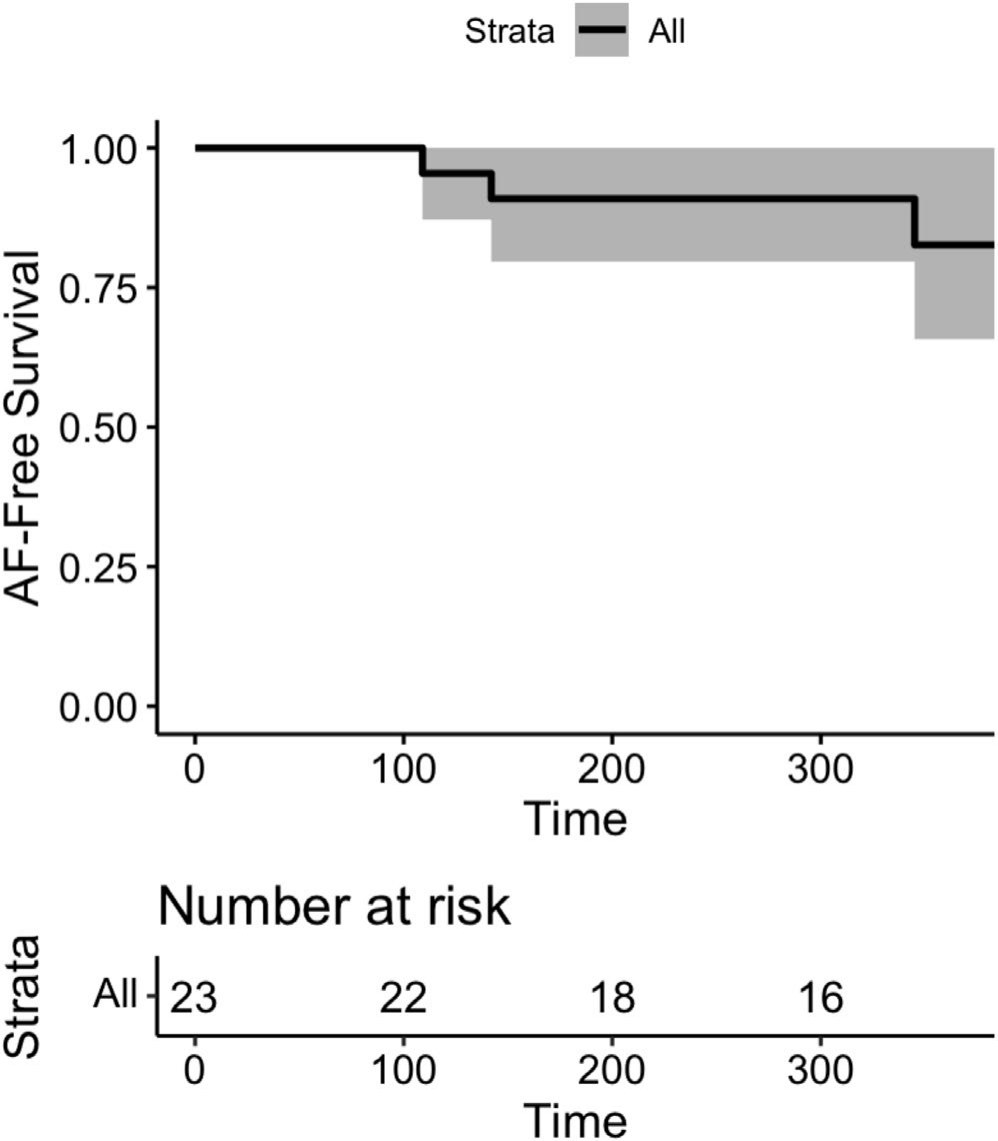
Kaplan–Meier analysis of atrial fibrillation (AF)–free survival following CSI‐guided ablation. Kaplan–Meier curve shows AF‐free survival over time in 23 patients who underwent catheter ablation with Complex Signal Identification (CSI) guidance. The shaded area represents the 95% confidence interval. The number of patients at risk at each time point (0, 100, 200, and 300 days) is listed below the *x*‐axis. AF, atrial fibrillation; CI, confidence interval; CSI, complex signal identification.

To evaluate the long‐term effectiveness of CSI‐guided ablation, we performed Kaplan–Meier survival analysis for arrhythmia‐free survival. The mean follow‐up duration was 326 ± 103 days. At 1 year, the arrhythmia‐free survival rate was 82.4%, as estimated from the Kaplan–Meier curve. (Figure 4).

## 4 Discussion

In this study, we evaluated the feasibility of using the CSI algorithm, newly implemented on the CARTO 3 platform, to identify non‐PV foci in patients undergoing AF ablation.

For quantitative analysis, CSI thresholds were fixed at 8.5; visual adjustment was used only for qualitative review during mapping, not for ROC evaluation. In clinical use, an adaptive threshold approach (initially set at 8.5, with minor upward or downward adjustment depending on the number of tagged regions) helped localize both left and right atrial non‐PV triggers.

The 1‐year arrhythmia‐free survival was 82%, suggesting that automated quantification of atrial electrogram fractionation can support the recognition of extra‐PV arrhythmogenic sources.

### 4.1 Clinical Implications of Targeting Non‐PV Foci

A growing body of evidence highlights the importance of non‐PV triggers as determinants of AF recurrence. In paroxysmal AF, durable rhythm control is far more likely when these triggers are absent or eliminated, whereas persistent ectopy is strongly associated with relapse [5]. In patients with concomitant left ventricular dysfunction, non‐PV foci are more prevalent, and their ablation has been linked with meaningful improvement in outcomes [15]. For persistent AF, comprehensive elimination of inducible triggers yields higher arrhythmia‐free rates compared with incomplete suppression (approximately three‐quarters versus one‐half) [7]. In a large multicenter registry, inducible non‐PV foci were found in the majority of cases, and their ablation was associated with superior clinical results [8].

Our present findings are aligned with these prior observations. When CSI was used in conjunction with provocation testing and activation mapping, we were able to successfully ablate relevant non‐PV drivers, leading to reduced recurrence. Furthermore, previous work with FAAM‐guided ablation demonstrated improved outcomes compared with conventional approaches, with an absolute benefit of about 15% in 1‐year success [10]. Non‐PV foci are particularly common in subgroups such as women, individuals with sinus node dysfunction, patients with prior cardiac surgery, atrial enlargement, or infiltrative cardiomyopathies including amyloidosis and sarcoidosis [16]. In these populations, accurate detection and targeted ablation of non‐PV foci may be especially critical.

### 4.2 Comparison of CSI With FAAM

Both CSI and FAAM provide objective, signal‐based approaches for detecting atrial regions exhibiting electrogram fractionation that may harbor AF drivers. CSI uses a machine learning–based algorithm embedded in CARTO 3 to assign fractionation scores (0–10) and display discrete tagged points directly on the 3D map, whereas FAAM (Rhythmia, Boston Scientific) applies a peak‐count criterion to generate color‐coded contiguous areas of fragmentation. Thus, while FAAM tends to visualize broader continuous regions, CSI highlights focal, pointwise complexity.

Indiscriminate ablation of all CSI‐positive sites could therefore create unnecessary lesions. In our cohort, a cutoff of 8.5 efficiently identified non‐PV foci; however, correlation with provoked ectopy and activation timing remained essential before lesion delivery. Algorithmically, CSI and FAAM differ in principle—machine‐learning classification versus deterministic peak counting—representing distinct but conceptually aligned approaches to quantifying atrial signal complexity. A detailed comparison is provided in Table S1.

### 4.3 Strategy for Selective Ablation

We did not perform ablation solely on the presence of elevated CSI scores. Instead, lesions were delivered only at sites where provocation with isoproterenol or ATP consistently induced ectopy and the earliest activation coincided with high CSI values. This selective approach helped to avoid unnecessary ablation of physiologically fractionated regions, such as those near the sinus and atrioventricular nodes, and reduced the risk of creating scar‐related atrial tachycardia [17, 18, 19]. In addition, anterior wall scarring has been associated with impaired left atrial function; therefore, a more conservative ablation strategy in this region is desirable [20]. Thus, CSI should be regarded as a complementary mapping tool that supports, but does not replace, provocation testing and activation mapping.

### 4.4 Limitations and Perspectives

Several methodological limitations should be acknowledged.

First, CSI output is rate‐ and rhythm‐dependent. During high right atrial pacing, longer cycle lengths reduced the number of fractionated sites, whereas faster pacing accentuated CSI scores. A pacing cycle of approximately 750–800 ms appeared optimal in our practice. Inadequate point density may underestimate electrogram complexity, emphasizing the need for uniform high‐density sampling. Certain anatomical sites (e.g., crista terminalis or coronary sinus ostium) can show fractionation despite non‐arrhythmogenicity, warranting cautious interpretation.

Second, the small sample size precluded multivariable analysis. Although ROC analysis consistently identified 8.5 as an effective threshold, this value should be regarded as exploratory, not definitive. Some non‐PV foci—particularly at septal or peri‐mitral sites—did not exhibit elevated CSI values, possibly due to insufficient sampling, suboptimal pacing cycle length, or local conduction anisotropy. These findings underscore that CSI is an adjunct rather than a substitute for provocation and activation mapping.

Third, this was a single‐center retrospective study with limited statistical power. Some degree of overfitting cannot be entirely excluded. CSI‐positive areas were estimated from point‐based surrogates, which may differ from true surface areas, particularly in regions with heterogeneous sampling density. Follow‐up relied mainly on ECGs and symptom‐driven evaluations, which may have underestimated silent recurrences. Finally, adjunctive ablations such as CTI or SVC isolation may have influenced the outcomes.

Future prospective multicenter studies with standardized CSI settings, systematic post‐ablation remapping, and comprehensive rhythm monitoring are warranted to validate these preliminary findings and to determine whether CSI integration improves long‐term ablation outcomes.

## 5 Conclusions

CSI on CARTO 3 provided feasible adjunctive guidance for identifying non‐pulmonary vein foci during atrial fibrillation ablation. Integration of CSI with provocation and activation mapping aided precise lesion delivery while avoiding unnecessary ablation. Although results were promising, the findings remain exploratory. Larger prospective studies are required to confirm the clinical value of CSI‐assisted mapping.

## Funding

The authors received no specific funding for this work.

## Ethics Statement

The research protocol received approval from the ethics committee at Kokura Memorial Hospital and adhered to the principles outlined in the Declaration of Helsinki.

## Consent

The need for written informed consent was waived by the institutional review board due to the retrospective nature of the study.

## Conflicts of Interest

The authors declare no conflicts of interest.

## Supporting information


**Table S1:** Comparison of ComplexSignal Identification (CSI) and Fractionated signal area in the atrial muscle (FAAM).

**Figure S1:** Workflow of CSI‐assisted mapping and ablation for non‐PV foci.
Workflow of the procedural sequence integrating Complex Signal Identification (CSI) into non–pulmonary vein (non‐PV) trigger ablation. The process comprises three main stages: (1) baseline mapping and pulmonary vein isolation (PVI) using CARTO 3 v8 with the CSI module activated under default settings; (2) provocation and identification of non‐PV foci using isoproterenol + adenosine triphosphate (ATP), requiring ≥ 10 atrial premature contractions (APCs) per minute to define active sites; and (3) CSI‐assisted ablation targeting the earliest activation site and adjacent regions with high CSI scores (≥ 8.5), followed by re‐provocation to confirm non‐inducibility. If the origin was located in the superior vena cava (SVC), SVC isolation (SVCI) was performed. The procedural endpoint was non‐inducibility of atrial fibrillation (AF), atrial tachycardia (AT), or repetitive ectopy after repeat provocation testing. APC, atrial premature contraction; CSI, Complex Signal Identification; PVI, pulmonary vein isolation; SVCI, superior vena cava isolation.
