# Supplementary material for: Relationship Between Complex Signal Identification and Non‐Pulmonary Vein Foci
Source: J Arrhythm. 2025 Nov 26;41(6):e70230. doi: 10.1002/joa3.70230 (PMC12657636; doi:10.1002/joa3.70230)
Supplement: Supplementary file 1 — Table S1: Comparison of ComplexSignal Identification (CSI) and Fractionated signal area in the atrial muscle (FAAM). [file JOA3-41-e70230-s002.docx]

**Table S1.** Comparison of ComplexSignal Identification (CSI) and Fractionated signal area in the atrial muscle (FAAM)

| **Feature** | CSI (CARTO v8,  Biosense Webster) | FAAM (Lumipoint, Rhythmia,  Boston Scientific) |
| --- | --- | --- |
| **Concept** | Machine learning–based  classification of atrial electrogram (EGM) complexity summarized as a  numeric score (0–10) per point. | Identifies **fractionated signal areas** using Lumipoint’s **peak slider (1–15)** to highlight fragmented electrograms; higher sliders (e.g., ≥7) isolate smaller, highly fractionated regions |
| **Primary input** | Bipolar EGMs acquired in sinus rhythm or atrial pacing with **Octaray** **catheter** | Bipolar EGMs acquired during sinus rhythm or atrial pacing with **ORION catheter** |
| **Output/visualization** | Point tags with per-point CSI score; no surface interpolation by default. | **Color-coded contiguous regions** representing FAAM, size inversely proportional to slider setting (e.g., 7.0 yields focused region). |
| **Thresholding** | User-selectable score threshold; in this study, ≥8.5 was used as an exploratory cutoff to prioritize targets. | Typically examined from **peak slider 7.0**, adjusted downward (7.0→6.0→5.0) until non-PV foci eliminated |
| **Dependence on**  **acquisition** | Sensitive to pacing rate and point density; higher rates increase detected fractionation; uniform  high-density sampling  recommended. | Dependent on **electrogram quality and density**; requires ≥5,000–10,000 mapping points for reliable FAAM delineation. |
| **Clinical purpose** | **Adjunctive localization**: helps identify slow-conduction zones adjacent to earliest activation; not a standalone ablation target. | **Substrate modification**: ablation of FAAM area until no inducible non-PV foci remain; median LA ablation area ≈10.5%, RA ≈5.5%. |
| **Lesion strategy** | Lesions at earliest activation and adjacent high-CSI points (selective). | Ablate FAAM area iteratively until non-PV foci non-inducible; avoid excessive area (>~10% LA). |
