# Supplementary material for: Relationship Between Complex Signal Identification and Non‐Pulmonary Vein Foci
Source: J Arrhythm. 2025 Nov 26;41(6):e70230. doi: 10.1002/joa3.70230 (PMC12657636; doi:10.1002/joa3.70230)
Supplement: Supplementary file 2 — Figure S1: Workflow of CSI‐assisted mapping and ablation for non‐PV foci. Workflow of the procedural sequence integrating Complex Signal Identification (CSI) into non–pulmonary vein (non‐PV) trigger ablation. The process comprises three main stages: (1) baseline mapping and pulmonary vein isolation (PVI) using CARTO 3 v8 with the CSI module activated under default settings; (2) provocation and identification of non‐PV foci using isoproterenol + adenosine triphosphate (ATP), requiring ≥ 10 atrial premature contractions (APCs) per minute to define active sites; and (3) CSI‐assisted ablation targeting the earliest activation site and adjacent regions with high CSI scores (≥ 8.5), followed by re‐provocation to confirm non‐inducibility. If the origin was located in the superior vena cava (SVC), SVC isolation (SVCI) was performed. The procedural endpoint was non‐inducibility of atrial fibrillation (AF), atrial tachycardia (AT), or repetitive ectopy after repeat provocation testing. APC, atrial premature contraction; CSI, Complex Signal Identification; PVI, pulmonary vein isolation; SVCI, superior vena cava isolation. [file JOA3-41-e70230-s001.pdf]

Baseline Mapping

- LA and/or RA electroanatomical mapping using CARTO 3 v8
- CSI module activated (default settings)
- PVI performed if not yet completed

Provocation of Non-PV foci

- Isoproterenol ± ATP administration
- ≥10 APCs/min required for mapping
- Identify earliest activation site
- If origin in SVC → SVC isolation added

CSI-Assisted Ablation

- Target = earliest activation ± adjacent high-CSI (≥8.5) sites
- Avoid indiscriminate ablation of broad CSI regions
- Re-provocation after ablation to confirm non-inducibility
- Endpoint = no provokable AF or repetitive ectopy
